# Supplementary material for: A redescription of Palaeogekko risgoviensis (Squamata, Gekkota) from the Middle Miocene of Germany, with new data on its morphology
Source: PeerJ. 2023 Jan 13;11:e14717. doi: 10.7717/peerj.14717 (PMC9841909; doi:10.7717/peerj.14717)
Supplement: Supplemental Information 2 [file peerj-11-14717-s002.docx]

**A redescription of *Palaeogekko risgoviensis* (Squamata, Gekkota) from the Middle Miocene of Germany, with new data on its morphology**

**Supplemental Data S2**

The revised scorings for *Palaeogekko risgoviensis* used in the phylogenetic analysis are as following:

Palaeogekko_risgoviensis ?0????-0??12?200?0?????????00??00????????????????????????????????????????????????????????????????????????????????????????????????????????????????????????????????????1000??0000-01002201120001-110?0000??10-0011000000000000000000????????????????????????????????????????????????????????????????????????????????-???????????????????????????????????????????????????????0?-??????1??????????????????????????0-?09{0 1}0?????????????????????????????????????????????????????????????????????????????????????????????????0?????????????????????????0?00???????????????????????????????????????0????????????????????????????????????????????????????????????????????????????????????????????????????????????????????????????????00?00---102?00000?0000000000?0?????????????????????????????????????????????????????????????????????????????????????????????????????????????000100?
